# The Action of Creosote and Carbolic Acid

**Published:** 1872-12

**Authors:** M. M’Carty


### The Action of Creosote and Carbolic Acid.

BY M. M'CARTY.

*Read before the Tennessee Dental Association.*

I propose in this paper to examine some of the statements made by members of this body upon creosote and carbolic acid.

One member said that it was a common impression that creosote destroyed the teeth, but that the opinion was wrong. Also that the vitality of a tooth could not be destroyed by creosote. Well, now let us examine into the nature and action of this agent, and see if the statements can be sustained. Creosote and carbolic acid belong to that class of agents known as escharotics. They have a strong chemical affinity for animal tissue, with which they form an eschar, scab or scale. This scab or scale is in every case deprived of its vitality, and being disorganized will be thrown off. The inflammation excited in the contiguous parts gives occasion to a secretion of pus between the living and dead parts, that causes the separation and throwing off of the dead. Now it is just this process by which the teeth are destroyed. The pulp of a tooth becomes exposed by decay. Acrid matter comes in contact with it, irritating and giving pain. An application of creosote is made, which relieves the pain by its narcotic effect; also by the protection the eschar affords it, by preventing air and substances in the mouth from coming in contact with it. But what is the result? There is as much of the pulp killed as the creosote united with, and will exfoliate sooner or latter, as the case is favorable or unfavorable to it. Then the pulp is exposed agaiu. The application is repeated time and again, as often as the tooth aches, every time eating a scale or scab off the pulp, until it has disorganized the whole, as also the nerve fibrils in the tubuli. By this time the decay has progessed to the destruction of the crown of the tooth. So they truthfully assert that they have destroyed their teeth by the use of creosote.

Another statement was, that carbolic acid has a conservative effect on the inside of a tooth, but a very destructive effect on the outside, upon the gums, and other mucous membrane. Its effects are always uniform; always devitalizing to the extent of its union with the animal tissue. He saw the effect on the outside, but did not investigate the inside, hence his statement. The eschar upon the mucous membrane is either rubbed off or washed off by the saliva. That of the pulp, less accessible, remains longer, and is dissolved off. That of the nerve fibrils within the tubuli of the dentine, still better protected, maintain their integrity for a still longer period, arresting decay until dissolution takes place.

Creosote nor carbolic acid form any compound with the lime salts; hence the eschar in the dentine of the nerve fibrils may have all the lime salts removed, by any chemical agent that has an affinity for it, and render the eschar porous, thereby destroying its protection to the part it covers.

Another statement, by another member, was, that creosote or carbolic acid formed with albumen an "insoluble compound." Now, if this is a fact, there need never be another tooth filled to arrest caries. They might be filled to strengthen them or improve their appearance, or to keep the mouth clean, by keeping the food from lodging and decaying in the cavities. In the use of creosote or carbolic acid in the cavity of decay in a tooth, if there is actually an "insoluble compound" formed, you have a perfect covering of the dentine, a barrier which the secretions of the mouth can not permeate.

The decomposition of food that may chance to get in the cavity, together with the thermal changes, will have no disintegrating effect upon it. But such is not the case, as every one will testify who has made applications of creosote or carbolic acid, and left the cavities open.

Whenever there is any substance brought in contact with the eschar, that has a stronger affinity for one of its elements than its elements have for each other, then will a solution of the eschar take place, and leave the part beneath exposed to further chemical action. The oral cavity is a most favorable place for chemical changes, on account of its temperature; the mucous acid; the alkaline saliva; also the variety of food and liquids that are so frequently passing through it. The chemical changes that are continually taking place in it make it quite certain that some element will be brought in contact with the eschar that will destroy its integrity.

By the decomposition in the cavity of decay, elements are set free in their nascent condition when their energy for union with other elements is greatest. Then will they seize hold on and break up other compounds. Hence we see that the eschar, the "insoluble compound," is not a perfect shield unless it be protected.

Why then should creosote and carbolic acid be used, and how should they be used?

They should be used by the dentist, because he can relieve pain with them, and make them conservative. His first use of them is to obtund sensitive dentine. Carbolic acid should be used for this, as it is more efficient in its action than creosote. It acts by devitalizing the ends of the nerve fibrils to the extent of its union with them, and also by its narcotic effect beyond the eschar.

They should be used also in the cavity prepared for filling, because the eschar formed is a perfectly fitting elastic cushion to the vital ends of the nerve fibrils.

It is probable too that the fibrils are not devitalized to as great a depth as they would be if left to heal without the escharotic application; consequently there will be more vitality preserved in the tooth. The same remarks are also applicable to the use of creosote and carbolic acid to the pulps of teeth, when the object is to preserve the vitality of an exposed pulp. After the cavity is prepared and dried, the escharotic is introduced to the pulp, an eschar is formed. Then if there can be put a filling on the eschar that will not press against the pulp, and all the other circumstances are favorable, the life of the pulp may be preserved. They are useful too in breaking up and changing the character of abscesses. They unite with the unhealthy granules and cells in the abscess, and substitute their own action to the contiguous parts in lieu of the existing pathological condition.

The eschar they form exfoliates, healthy cell formation sets in, and continues until the fistulous opening is filled.

They may be used with great advantage also between the gums and the teeth, where there is absorption of the alveolus, after the removal of salivary calculus.

Pure creosote can only be made from wood tar. V.

manufacture is attempted with coal tar it is more or less contaminated with carbolic acid. It has a composition of $C_{14}$ $H_8$ $O_2$

Carbolic acid is made from coal tar. It being the product that passes over at between three and four hundred degrees Fahrenheit. It has a composition of $C_{12}$ $H_5$. O. HO.; when first made it is a liquid at 90°, and all crystalized at 70° Upon exposure to the air it deliquesces, and will not all crystalize again, but at low temperatures there will be a part of it crystalized. After exposure for a while in the summer time it is all liquid; when the cold weather comes on part of it formed into transparent needles. M. M'CARTY.

